# Editorial: Psychometrics in psychiatry 2022: anxiety and stress disorders

**DOI:** 10.3389/fpsyt.2023.1352047

**Published:** 2024-01-05

**Authors:** Wenjie Duan, Jingying Wang, Inês A. Trindade, Andras N. Zsido

**Affiliations:** ^1^Social and Public Administration School, East China University of Science and Technology, Shanghai, China; ^2^Center for Health and Medical Psychology (CHAMP), School of Behavioural, Social and Legal Sciences, University of Örebro, Örebro, Sweden; ^3^Faculty of Humanities and Social Sciences, Institute of Psychology, University of Pécs, Pécs, Hungary

**Keywords:** scale development, validation, machine learning, mental health, non-WEIRD population

## Introduction

As prevalent mental health disorders, anxiety and stress disorders exert a significant impact on global health-related burdens as well as individual wellbeing. This impact has been aggravated by the COVID-19 pandemic and international conflicts, such as the Russo-Ukrainian War (1). Notably, the pandemic has led to a 27.6% and 25.6% upturn in the global prevalence of major depressive disorder and anxiety disorders respectively (2). During the pandemic, quarantine and social isolation have been found to intensify the anxiety levels of residents (3). According to Li et al. (4), Santomauro et al. (2), and Lábadi et al. (5), the pandemic has disproportionately affected women, elderly, children, and adolescents.

The enhancement of outcomes in stress and anxiety heavily depends on operationally defined and measured related concepts. For instance, in order to comprehend the barriers to individual wellbeing amid the COVID-19 pandemic, Li et al. (6) and Choi et al. (7) have developed tools to evaluate fear and perceived stigma. However, the validity of self-reported psychometric tools, long-standing due to their subjectivity, has been a point of concern (8, 9). Moreover, most measures for stress and anxiety disorders have been developed and validated in Western, Educated, Industrialized, Rich, and Democratic (WEIRD) populations. These include, among others, the Perceived Stress Scale (10), Patient Health Questionnaire (11), and Depression Anxiety Stress Scales (12). However, non-WEIRD and socially disadvantaged groups, such as children and individuals with low income or less education, face heightened risks of stress and anxiety (13).

In response to these concerns, this Research Topic aims to consolidate research providing critical insights into new psychometrics in psychiatry. Specifically, it intends to pinpoint limitations in our existing understanding of the differences between clinimetric and psychometric measures, introduce new concepts related to stress and anxiety disorders, adapt validated scales across various contexts, and identify other stress- and anxiety-related determinants in non-WEIRD populations.

## Structure and contribution of the Research Topic

In response to our call for advancing knowledge on reliable and valid methods for measuring anxiety and stress, this Research Topic incorporated six manuscripts (Table 1). One manuscript examines the disparities between psychometric and clinimetric approaches in measurement, based on the assessment of arachnophobia. Another introduces the newly-developed Anxiety and Fear of COVID-19 Assessment Scale (AMICO) Pregnant Scale, designed to evaluate the fear and anxiety experienced by pregnant women during the pandemic. Additionally, three manuscripts explore the psychometric properties of anxiety-related concepts across various cultural contexts. Finally, a cross-sectional study identifies factors associated with depression, behavioral problems, and cognitive functions in Chinese adolescents.

**Table 1:** Summary of contributions to the Research Topic.

**No**	**Author**	**Title**	**Purpose**	**Views**
1	Dong et al.	*Determinants of Depression, Problem Behavior, and Cognitive Level of Adolescents in China: Findings from a National, Population-Based Cross-Sectional Study*	This study aimed to assess the associated factors for adolescent depression, problem behavior and cognitive level in China.	1,068
2	Schäfer et al.	*The Perceived Stress Scale 2 and 2: A Two-Factorial German Short Version of The Perceived Stress Scale*	While the Perceived Stress Scale (PSS) is a widely used questionnaire, a German short version of the scale is not yet available. In the current study, we developed such a short version using a machine learning approach for item reduction to facilitate the simultaneous optimization of multiple psychometric criteria.	1,668
3	Landová et al.	*Toward a Reliable Detection of Arachnophobia: Subjective, Behavioral, and Neurophysiological Measures of Fear Response*	This study aimed to compare scores on questionnaires and image evaluation to the objective measurements of the behavioral approach test (BAT) and the neurophysiological effect of spiders extracted from fMRI measurements. The objective was to explore how reliably subjective statements about spiders and physiological and behavioral parameters discriminate between phobics and non-phobics, and what are the best predictors of overall brain activation.	1,333
4	Ionio et al.	*The Italian Language Postpartum Specific Anxiety Scale [PSAS-IT]: Translation, Psychometric Evaluation, and Validation*	This current study aimed to translate and validate the Postpartum Specific Anxiety Scale [PSAS] into the Italian language, and to validate the tool for its use in detecting anxiety specific to motherhood.	736
5	Muñoz-Vela et al.	*Adaptation and Psychometric Study of the Scale for the Measurement of Fear and Anxiety of COVID-19 Disease in Pregnant Women (AMICO_Pregnant)*	The aim of this research was to adapt and explore the psychometric properties of a specific scale to assess the levels of fear and anxiety of COVID-19 disease in pregnant women.	512
6	Zhang et al.	*Factor Structure and Psychometric Properties of the Chinese Version of the Odor Awareness Scale*	The Odor Awareness Scale (OAS) is a questionnaire that assesses individual differences in awareness of odors in the surrounding environment, which has been shown to be associated with affective symptoms in recent researches. This study aimed to investigate the factor structure and validate the psychometric properties of the Odor Awareness Scale.	620

### Assessment of clinimetric and psychometric measures

Arachnophobia, as a common anxiety disorder, can significantly impact an individual's quality of life. In an effort to discern the discrepancies between subjective (e.g., self-reported questionnaires) and objective (e.g., behavioral approach tests [BAT] and functional Magnetic Resonance Imaging [fMRI]) assessments of arachnophobia, Landová et al. conducted a study involving 30 arachnophobic participants and 32 non-phobic controls, all of whom had been previously diagnosed through clinical interviews. The study employed subjective fear response measures such as the Spider Questionnaire (SPQ), Snake Questionnaire, Disgust Scale-Revised, and a subjective emotional assessment of spider-related visual stimuli. On the other hand, the behavioral fear response was gauged using the BAT, which quantified the participants' level of avoidance behavior toward live spiders. Furthermore, neurophysiological responses were measured using fMRI, resulting in the formulation of a Spider Fear Index. The researchers found that subjective assessments, including the SPQ, BAT, and emotional evaluations of visual stimuli, were not only easy to administer and cost-effective, but also demonstrated reliability as measures of arachnophobia.

### New scale validation

During the COVID-19 pandemic, Muñoz-Vela et al. identified heightened vulnerability and specific challenges faced by marginalized social groups, including pregnant women, who exhibited increased risks of anxiety and stress-related disorders. To accurately assess these psychological dimensions, the researchers adapted the Anxiety and Fear of COVID-19 Assessment Scale (AMICO), creating a specialized version for pregnant women, dubbed AMICO_Pregnant. This novel scale was psychometrically evaluated in a study involving 1,013 pregnant women residing in Spain. The analysis revealed two distinct factors across 16 items-fear and anxiety-both demonstrating strong model fit parameters. Furthermore, the scale exhibited high internal consistency, indicative of its reliability as a measure of pandemic-related fear and anxiety among pregnant individuals.

### Evaluating cross-context adaptations

The Perceived Stress Scale (PSS) is a prominent instrument for measuring subjective stress; however, its short version's adaptation into German requires further examination, as indicated by Schäfer et al.. Their study, which included a sample of 1,437 participants from an online panel, not only assessed traditional psychometric properties but also incorporated an innovative machine-learning technique-ant-colony-optimization (ACO) algorithms-to refine the item selection process. In addition to these methodologies, the authors utilized network analysis, an emerging psychometric tool, to evaluate the construct validity of the revised scale. Their findings identified a bifactor model with dimensions labeled “helplessness” and “self-efficacy,” both demonstrating robust psychometric characteristics, such as construct validity, internal consistency, and test-retest reliability.

Adaptation difficulties during the postpartum period are commonly reported among mothers, yet previous measures have either overlooked the distinctive characteristics of this stage or failed to acknowledge the continuum between pregnancy-related anxiety and anxiety experienced at other stages of life. Ionio et al. emphasized the importance of the Postpartum Specific Anxiety Scale (PSAS) as the sole instrument designed to assess postpartum anxiety. However, the Italian version of the PSAS requires validation. In a study involving a cross-sectional sample of 457 new mothers, the authors demonstrated that the Italian adaptation of the PSAS retains the psychometric properties of the original scale, exhibiting satisfactory construct and convergent validity, as well as internal consistency. Furthermore, multi-group confirmatory factor analysis suggests that the PSAS is psychometrically invariant across different cultural contexts, specifically between Italy and the United Kingdom.

Zhang et al. adapted the Odor Awareness Scale for the Chinese population, offering evidence of its construct, convergent, and concurrent validity, as well as its internal consistency and test-retest reliability. Employing a sample of 978 college students, the authors demonstrated that their three-factor adaptation of the Chinese Odor Awareness Scale yielded favorable model fit indices. The scale's subscales assess odor sensitivity, impact, and attention, respectively. Moreover, the scale exhibited positive correlations with individual body odor-sniffing behaviors and anxiety levels.

### Survey design paper

Dong et al. analyzed survey data from the 2018 China Family Panel Studies (CFPS), examining a cohort of 2,584 adolescents between the ages of 10 and 15 in China. Employing a quantitative methodology, the researchers utilized *t*-tests, one-way analysis of variance (ANOVA), and multivariate linear regression to identify factors correlated with depression, problematic behaviors, and cognitive outcomes. The findings underscored that determinants of mental health were primarily associated with school-related factors and the depressive symptoms of parents. Moreover, determinants of problematic behavior included lower maternal education, the adolescents' poor health, elevated academic stress, and parental depression. Regarding cognitive outcomes, poor academic performance and paternal depression emerged as significant predictors.

## Conclusion

The increasing global prevalence of anxiety and stress disorders requires a comprehensive and inclusive methodology for their assessment and treatment. This Research Topic collated an assortment of contributions on measurement tools and definitions of stress- and anxiety-related concepts, enabling a review of evidence for different procedures and tools. This understanding is essential for addressing conditions and processes that impede the functioning and wellbeing of individuals, groups, and institutions. Nevertheless, it must be highlighted that there is a pressing need for more systematic or meta-analytic review studies focussing on psychometric scales, given their potential to investigate a broad spectrum of diagnostic accuracy measures in individuals of diverse demographic characteristic (14, 15). Despite these gaps, we remain hopeful that this compilation of articles will stimulate scholars to explore new territories and offer a comprehensive guide for future research endeavors.

## Author contributions

WD: Conceptualization, Writing – original draft, Writing – review & editing. JW: Conceptualization, Writing – original draft, Writing – review & editing. IT: Writing – review & editing. AZ: Writing – review & editing.
